# Composite Mantle Cell Lymphoma and Chronic Lymphocytic Leukemia/Small Lymphocytic Lymphoma: A Diagnostic and Therapeutic Challenge

**DOI:** 10.1002/jha2.70222

**Published:** 2026-01-30

**Authors:** Matthew T. Ye, Yaling Yang, M. James You

**Affiliations:** ^1^ Department of Hematopathology The University of Texas MD Anderson Cancer Center Houston Texas USA; ^2^ Vanderbilt University School of Medicine Nashville Tennessee USA; ^3^ Department of Pathology City of Hope National Medical Center Duarte California USA

**Keywords:** composite, CLL/SLL, MCL

1

A 74‐year‐old woman with a history of monoclonal B‐cell lymphocytosis presented with inguinal lymphadenopathy. Lymph node biopsy revealed mantle cell lymphoma (MCL). She subsequently sought treatment at our hospital. PET/CT showed lymphadenopathy above and below the diaphragm. Bone marrow biopsy demonstrated multiple atypical lymphoid aggregates, involving 20% of the marrow. Aspirate smear showed increased lymphocytes with irregular nuclear contours, condensed chromatin, and scant cytoplasm. Immunostains revealed that the lymphoid aggregates were composed predominantly of CD20+, PAX5+ B‐cells that aberrantly co‐expressed CD5. Surprisingly, only the cells in the central region of the aggregates were positive for cyclin D1 and SOX11, consistent with MCL, while cells in the periphery expressed CD23 and LEF1, consistent with chronic lymphocytic leukemia/small/lymphocytic lymphoma (CLL/SLL) (Figure 1).

**FIGURE 1:**
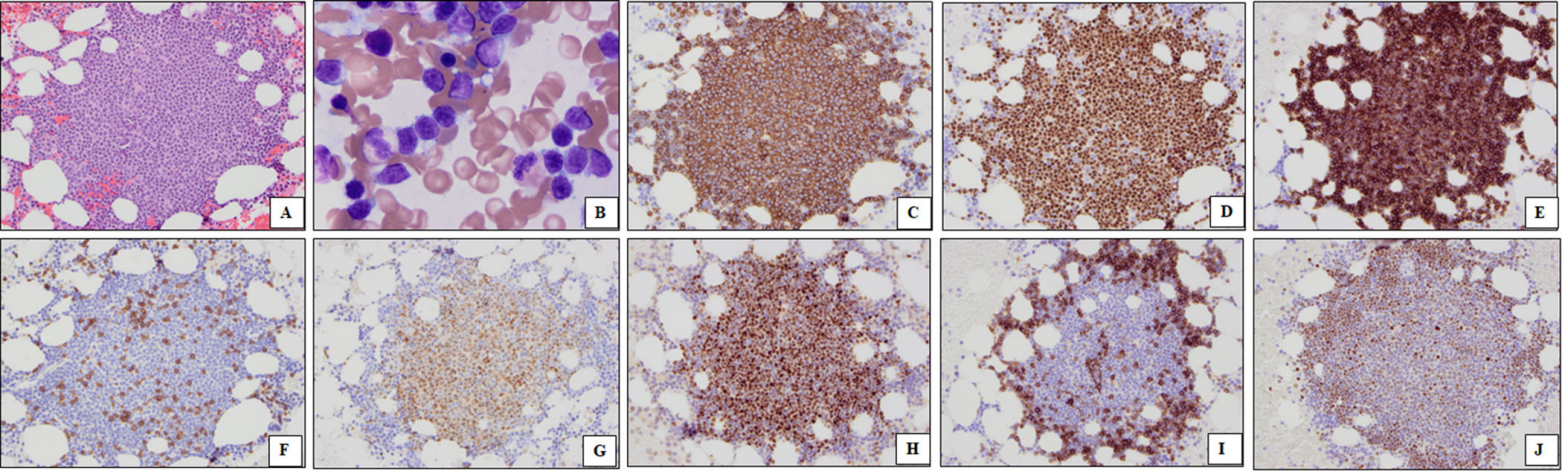
Morphologic findings. Histologic sections of the bone marrow clot section show atypical lymphoid aggregates (A, H&E, 200×). Aspirate smears show that the lymphoid cells are predominantly of small to intermediate size with condensed chromatin and scant cytoplasm (B, Wright–Giemsa, 400×). Immunostains show that the lymphoid cells are CD20+ (C, 200×) and PAX5+ (D, 200×) and aberrantly co‐express CD5 (E, 200×), with a few admixed reactive CD3+ T‐cells (F, 200×). The lymphoid cells in the central regions of the aggregates are positive for cyclin D1 (G, 200×) and SOX11 (H, 200×), consistent with MCL, while cells in the peripheral rims express CD23 (I, 200×) and LEF1 (J, 200×), consistent with CLL/SLL.

Flow cytometry identified two distinct B‐cell populations, both CD5‐positive. One population expressed dimmer CD19, CD43 (partial), and monotypic kappa light chain, but lacked CD23 and CD200, consistent with MCL. The other showed brighter CD19, CD23, CD43, CD200, and monotypic lambda light chain, consistent with CLL/SLL (Figure 2). Cytogenetic analysis showed 46, XX[20]. FISH was positive for *IGH* *::CCND1* rearrangement (8.5%), *ATM* gene deletion (96.5%), and loss of 13q14.3 (15.5%). Molecular testing detected monoclonal IGH rearrangements and *IGH::CCND1* fusion. No mutation was identified by targeted next‐generation sequencing.

**FIGURE 2:**
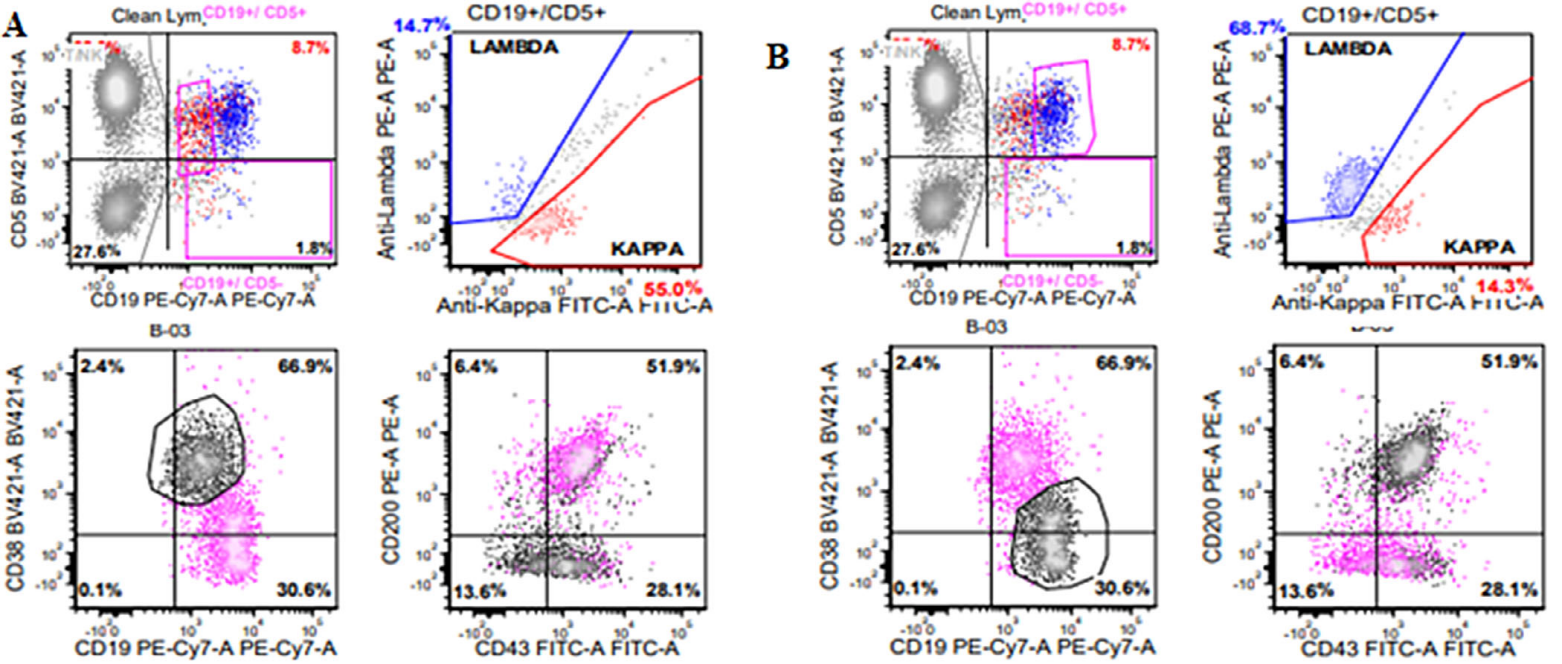
Flow cytometry identified two distinct B‐cell populations, both CD5‐positive. One population expressed dimmer CD19, CD43 (partial), and monotypic kappa light chain, but lacked CD200, consistent with MCL (A). The other showed brighter CD19, CD43, CD200, and monotypic lambda light chain, consistent with CLL/SLL (B).

The patient received acalabrutinib and rituximab, achieving clinical and morphologic remission within 7 months. Unfortunately, she passed away 2 years later from an unknown cause.

Composite lymphomas, defined as the coexistence of two distinct lymphoid neoplasms within the same anatomical site, are rare, accounting for < 5% of all lymphomas. Most cases involve a combination of classical Hodgkin lymphoma with a non‐Hodgkin lymphoma [1]. Composite lymphomas composed of two mature B‐cell neoplasms are particularly uncommon [2], with concurrent MCL and CLL/SLL being exceptionally rare [3, 4].

MCL is an aggressive B‐cell lymphoma characterized by overexpression of cyclin D1 due to the t(11;14)(q13;q32) translocation. In contrast, CLL/SLL is a slow‐growing B‐cell lymphoma with a distinct immunophenotype and variable clinical behavior. This case highlights the diagnostic complexity of composite MCL and CLL/SLL in a single bone marrow. Accurate diagnosis relies on integrated evaluation, including histology, immunohistochemistry, flow cytometry, cytogenetics, and molecular studies. The detection of distinct light chain restriction patterns (kappa for MCL; lambda for CLL/SLL) suggests these are distinct disease processes arising from separate neoplastic clones rather than divergent evolution from a common precursor.

From a clinical perspective, the coexistence of an aggressive lymphoma (MCL) with an indolent one (CLL/SLL) introduces complexity in treatment decisions. Treatment for CLL/SLL is often conservative, especially in asymptomatic or early‐stage cases, while MCL typically requires more aggressive therapy. Management often prioritizes the more aggressive component, though therapy must be tailored to disease burden and molecular risk features.

In conclusion, this case illustrates the diagnostic intricacies and clinical implications of composite lymphomas involving MCL and CLL/SLL, reinforcing the importance of an integrated diagnostic approach and appropriate clinical management. The case contributes to the limited body of literature on dual B‐cell lymphomas and raises important questions about clonal evolution, disease pathogenesis, and personalized therapy.

## Author Contributions

All authors contributed to the paper's conception and design. Clinical and laboratory data were collected by Y.Y. and M.J.Y. The manuscript was written by M.T.Y. All authors read and approved the final manuscript.

## Funding

The authors have nothing to report.

## Ethics Statement

This study was conducted according to an institutional review board‐approved laboratory protocol and in accordance with the Declaration of Helsinki.

## Consent

Informed consent was obtained from the patient.

## Conflicts of Interest

The authors declare no conflicts of interest.

## Data Availability

The data from this study will be available upon reasonable request.
